# Structural Analysis and Mutant Growth Properties Reveal Distinctive Enzymatic and Cellular Roles for the Three Major L-Alanine Transaminases of *Escherichia coli*

**DOI:** 10.1371/journal.pone.0102139

**Published:** 2014-07-11

**Authors:** Esther Peña-Soler, Francisco J. Fernandez, Miguel López-Estepa, Fernando Garces, Andrew J. Richardson, Juan F. Quintana, Kenneth E. Rudd, Miquel Coll, M. Cristina Vega

**Affiliations:** 1 Centro de Investigaciones Biológicas, Consejo Superior de Investigaciones Científicas (Spanish National Research Council, CSIC), Madrid, Spain; 2 Institute for Research in Biomedicine (IRB Barcelona), Barcelona, Spain; 3 Institut de Biologia Molecular de Barcelona (IBMB-CSIC), Barcelona, Spain; 4 The Scripps Research Institute, La Jolla, California, United States of America; 5 University of Miami Miller School of Medicine, Miami, Florida, United States of America; Rochester Institute of Technology, United States of America

## Abstract

In order to maintain proper cellular function, the metabolism of the bacterial microbiota presents several mechanisms oriented to keep a correctly balanced amino acid pool. Central components of these mechanisms are enzymes with alanine transaminase activity, pyridoxal 5′-phosphate-dependent enzymes that interconvert alanine and pyruvate, thereby allowing the precise control of alanine and glutamate concentrations, two of the most abundant amino acids in the cellular amino acid pool. Here we report the 2.11-Å crystal structure of full-length AlaA from the model organism *Escherichia coli*, a major bacterial alanine aminotransferase, and compare its overall structure and active site composition with detailed atomic models of two other bacterial enzymes capable of catalyzing this reaction *in vivo*, AlaC and valine-pyruvate aminotransferase (AvtA). Apart from a narrow entry channel to the active site, a feature of this new crystal structure is the role of an active site loop that closes in upon binding of substrate-mimicking molecules, and which has only been previously reported in a plant enzyme. Comparison of the available structures indicates that beyond superficial differences, alanine aminotransferases of diverse phylogenetic origins share a universal reaction mechanism that depends on an array of highly conserved amino acid residues and is similarly regulated by various unrelated motifs. Despite this unifying mechanism and regulation, growth competition experiments demonstrate that AlaA, AlaC and AvtA are not freely exchangeable *in vivo*, suggesting that their functional repertoire is not completely redundant thus providing an explanation for their independent evolutionary conservation.

## Introduction

Amino acid homeostasis maintains and regulates the balance of cellular amino acid pools in bacterial microbiota. This balance is not only a key metabolic prerequisite for bacterial growth but also contributes to human host amino acid homeostasis by exchanges through the intestinal mucosa [1]. Alanine plays a crucial role in amino acid homeostasis because it is abundant (second only to aspartate and glutamate in cellular concentration) and plays an important role in sugar and amino acid biosynthetic and catabolic pathways through reversible transamination reactions, thereby linking crucial metabolic networks like fermentation (*via* pyruvate) [2], [3] and nitrogen metabolism (*via* aspartate and glutamate) [4]. At the crossroads of these pathways, several partially overlapping enzyme activities collectively known as glutamic-pyruvic acid transaminases (GPTs) or alanine transaminases (EC 2.6.1.2) participate in functions such as alanine *de novo* biosynthesis, which is essential for the bacterial cell viability (since both L-alanine and its D isomer are building blocks of the cell wall) [5], and alanine degradation, depending on metabolic and environmental conditions**.** The central metabolic functions performed by GPTs are not restricted to bacteria but they are widely shared across archea and eukaryotes. In humans, for instance, the two alanine aminotransferase isoenzymes ALT1 and ALT2 have tissue-specific distributions (being present in kidney, liver and cardiac and skeletal muscle) and their serum levels together with those of aspartate aminotransferase (AAT) are used as diagnostic markers in hepatic, metabolic and cardiovascular dysfunctions [6]–[8].

**Figure 1:**
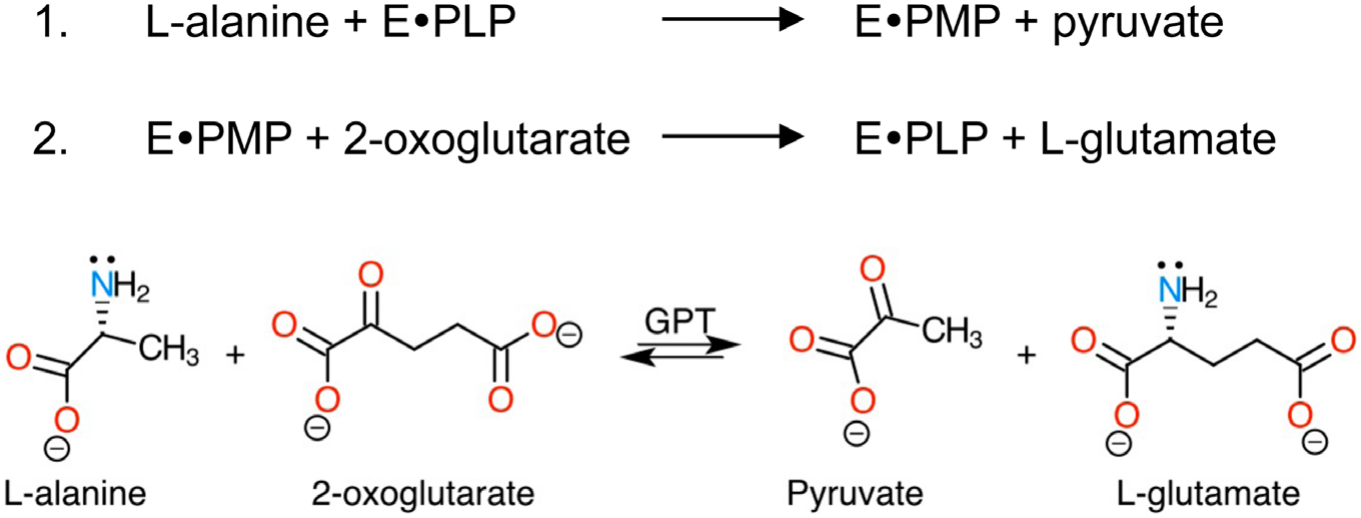
Scheme of the net reaction catalyzed by alanine transaminase (glutamic acid-pyruvic acid transaminase, GPT). In the first half-reaction (1) L-alanine is converted to pyruvate with the concomitant conversion of the Lys-PLP Schiff-base linked cofactor to free Lys and PMP (in AlaA, the catalytic lysine residue is Lys240). In the second and last half-reaction (2), a molecule of 2-oxoglutarate is converted to L-glutamate and PMP is recycled back to the enzyme’s resting state cofactor (Lys240-PLP). In the net reaction scheme, a lonely electron pair is shown beside the reactive amine groups of L-alanine and L-glutamate. In the case of valine-pyruvate transaminase (AvtA), the incoming oxo acid is 3-methyl-2-oxobutanoate yielding L-valine as the corresponding α-amino acid instead of L-glutamate.

All GPTs thus far characterized are pyridoxal 5′-phosphate (PLP, or vitamin B_6_)-dependent enzymes of fold type I [9] that catalyze the two sequential reactions that interconvert alanine and 2-oxoglutarate into pyruvate and glutamate, respectively, via the formation of covalent adducts of the incoming substrates with the PLP cofactor (**Figure 1** and **Figure S1** in **File S1**). As such, PLP-dependent aminotransferases are assumed to share a common enzymatic mechanism for alanine transamination that implies cofactor recycling from a covalently linked internal aldimine with an enzyme’s key catalytic lysine residue (Lys-PLP) to covalent substrate adduct intermediates termed external aldimines (**Figure S1** in **File S1**) [4], [10]–[12]. Factors such as the orientation of the scissile bond and the electron repartition within the resonance system of the covalent adduct are heavily influenced by the array of interactions established between the various cofactor forms and the active site residues, which ultimately determine the course of the reaction pathway toward transamination, decarboxylation, desulfination, elimination or aldol cleavage [4], [11], [13]. The degree of versatility afforded by the chemistry of PLP-dependent enzyme catalyzed reactions has indeed motivated the use of these catalysts as scaffolds for enzyme redesign and protein engineering studies of aspartate and aromatic amino acid transaminases [10], [14]–[16].

Recently, the alanine transaminases AlaA and AlaC [17], [18] and the valine-pyruvate aminotransferase AvtA (2.6.1.66) [18], [19] have been genetically identified as the three major alanine aminotransferases of the model organism *Escherichia coli* K-12 [17]. In the last years, classical approaches [17] and systems biology and transcriptomics methods [20] have been applied to identify the alanine transaminase-encoding genes and to elucidate the transcription regulatory network that controls their gene expression. Those studies highlight the biological redundancy built in amino acid metabolism and, in particular, alanine transamination, to ensure proper functioning of the metabolic network in adverse circumstances or in changing environments [21], [22]. The more or less relaxed substrate specificity (promiscuity) of these enzymes explains that about eleven different proteins can catalyze the transamination of alanine in *E. coli* [17] although only the triple gene deletion mutant (Δ*alaA* Δ*alaC* Δ*avtA)* has been reported to show a suboptimal phenotype [17]. This degree of redundancy poses the challenge of deciding which enzymes are dedicated alanine transaminases and which are the specific effects of their function that have allowed the three recently characterized major alanine aminotransferases AlaA, AlaC and AvtA to be conserved despite their overlapping reaction profile [17].

Here we describe the 2.11-Å resolution crystal structure of full-length AlaA of *E. coli* in complex with a substrate-mimicking acetate molecule locked into the active site in the binding pocket of the α-carboxylate group of the incoming substrates, alanine and 2-oxoglutarate. Despite the fact that several crystal structures of alanine transaminases have been deposited in the Protein Data Bank (PDB), the structure of AlaA provides the first glimpse of a completely structured bacterial GPT active site poised for catalysis. In addition, we assembled homology models of *E. coli* AlaC and AvtA, thereby facilitating the comparison of the active site configurations of these enzymes to shed light on their partially overlapping functions and pointing to a common reaction mechanism that could be universally shared by all alanine aminotransferases. In agreement with the imperfect overlap of their substrate specificities and reactivity and their evolutionary conservation, the genes encoding each of these enzymes were found not to be completely redundant in conditions resembling the intestinal environment or under growth competition under standard culture conditions, indicating that, at variance with today’s accepted view, even alanine transaminase single-gene deletion strains can undergo substantial reductions in growth rates and survival.

## Materials and Methods

### Cell growth

Bacterial cell cultures were grown at 37°C in four distinct media either statically (no agitation, anaerobic or microaerobic conditions) or aerobically (vigorous shaking, 220 rpm); statically grown cultures were assumed to better approximate the oxygen-limiting adhesive growth characteristics of bacteria onto the human intestinal mucosa than aerobic cultures. The media used and their compositions were as follows. LB is standard Luria-Bertani broth. DMEM stands for Dulbecco’s modified Eagle medium (Gibco ref. 41965). M9 minimal medium was used as such or supplemented with 10 mM L-alanine; the composition of M9 included 12.8 g/l Na_2_HPO_4_·2H_2_O, 3 g/l KH_2_PO_4_, 0.5 g/l NaCl, 1 g/l NH_4_Cl, 0.24 g/l MgSO_4_, 0.01 g/l thiamine, pH 7.4, and 0.4% (w/v) D-glucose as carbon source. Overnight cultures contained either no antibiotics or kanamycin (50 µg/ml).

### Bacterial strains and plasmids

The strains and plasmids used in this study are described in **Table 1**. Plasmid preparations and subcloning of the *alaA* gene was performed in *E. coli* XL1-Blue (Stratagene) and recombinant expression was performed in *E. coli* BL21(DE3). All strains used for growth rate measurements and competition experiments were derivatives of the reference wild-type (WT) *E. coli* K-12 strain BW25113 and comprised the three knockout (KO) strains JW2287 (Δ*alaA*), JW2376 (Δ*alaC*) and JW5652 (Δ*avtA*). These strains were obtained from the Keio collection of single-gene deletion strains made available from the Coli Genetic Stock Center (CGSC) [23], [24]. The three Keio strains carry a kanamycin resistance cassette that allows for antibiotic selection against the WT strain. All three strains are isogenic with BW25113 and have not acquired any hyper-motility mutations [25].

**Table 1:** Bacterial strains and plasmids.

	Relevant genotype or phenotype	Source
**Strains**		
XL1-Blue	F^−^::Tn10 proA^+^B^+^ lacIq Δ (lacZ) M15/recA1 endA1 gyrA96 (Nalr) thi hsdR17 (r_K_^−^m_K_^+^) glnV44 relA1 lac	Stratagene
BL21(λDE3)	E. coli B ompT hsdS(r_B_^−^m_B_^−^) gal dcm (DE3)	Novagen
BW25113	F^−^, Δ (araD-araB)567, ΔlacZ4787(::rrnB−3), λ^−^, rph−1, Δ (rhaD–rhaB)568, hsdR514	Keio collection
JW2287	BW25113 ΔalaA772::kan	Keio collection
JW2376	BW25113 ΔalaC778::kan	Keio collection
JW5652	BW25113 ΔavtA775::kan	Keio collection
**Plasmids**		
pAlaA	pT7::His_6_-AcTev-AlaA Kan^R^	This work

The pETM11 vector encoding an N-terminal hexahistidine (His) tag and a tobacco-etch-virus TEV protease cleavage site was used to construct pAlaA. A 1242-bp PCR fragment was amplified from *E. coli* XL1-Blue genomic DNA using primers 5′-AGCTATGGTCTCCCATGTCCCCCATTGAAAAATCCAG-3′ and 5′-ACTGCTCGAGTTACAGCTGATGATAACCAGAAAGG-3′. It was then digested with BsaI and BamHI and ligated into NcoI/BamHI-digested vector with the Rapid Ligation Kit (Roche). The resulting pAlaA expression plasmid was verified by sequencing.

### Expression and purification of *E. Coli* AlaA

*E. coli* BL21(DE3) was transformed with pAlaA and plated on LB agar plates supplemented with 50 µg/ml kanamycin and 2% (w/v) D-glucose. An overnight starter culture incubated with a single colony was diluted 1∶50 in a two-liter flask with Superior Broth (AthenaES) and growth was continued at 37°C with shaking until reaching an absorbance at 600 nm (OD_600_) of 0.8. The culture was then transferred to 20°C and induced one hour later with 1 mM isopropyl-β-D-thiogalactopyranoside (IPTG) for 20 h. Cells were harvested by centrifugation and the cell pellet was flash-frozen in liquid nitrogen and stored at −80°C until further use.

All purification steps were performed at 4°C. The cell pellet was resuspended in 4× the cell wet weight (20 ml) in lysis buffer consisting of 50 mM Tris-HCl, 500 mM NaCl, 20 mM imidazole, pH 8.0, 30 µg/ml lysozyme, 20 µg/ml DNase I, 10 mM MgSO_4_, 2 mM β-mercaptoethanol, 1 mM phenylmethylsulfonyl fluoride and 1 tablet EDTA-free Complete protease inhibitor cocktail. Cell breakage was achieved by ten cycles of sonication (30-s burst with 2-min breaks) and the crude lysate was clarified by two centrifugations at 60,000 *g* for 30 min at 4°C and filtration through a 0.45-µm membrane. The clarified lysate was then purified by metal-affinity chromatography using a 5-ml HisTrap column (GE Healthcare) followed by size-exclusion chromatography on a Superdex 200 10/300 GL column (GE Healthcare). A peak containing pure His-tagged AlaA eluted with an estimated molecular mass of 90,000 Da, corresponding to a dimer. His-tagged AlaA was concentrated in storage buffer (20 mM Tris-HCl, 100 mM NaCl and 2 mM dithiothreitol [DTT], pH 8.0). Protein concentration was determined by absorbance at 280 nm.

### Crystallization and X-ray data acquisition

For crystallization purposes, His-tagged AlaA was concentrated to 10 mg/ml in storage buffer and vapor-diffusion sitting-drop experiments were set up at 20°C by mixing 1 µl protein with 1 µl crystallization solution over 0.5 ml of mother liquor. Suitable crystals appeared in 0.25 M lithium acetate and 22% (w/v) polyethylene glycol (PEG) 3350, reaching a final size of 0.3×0.3×0.45 mm after one week. For cryoprotection, crystals were briefly immersed in mother liquor containing 15% (v/v) sterile glycerol and immediately flash-frozen in liquid nitrogen. X-ray diffraction data were collected at the ID29 beamline of the European Synchrotron Radiation Facility (ESRF, Grenoble, France). A complete data set to a maximum resolution of 2.11 Å was indexed and integrated using XDS [26] and then scaled and merged with SCALA [27]. Data collection statistics are summarized in **Table 2**. Crystals belonged to the orthorhombic space group *C*222_1_ with unit-cell parameters *a* = 59.8 Å, *b* = 152.1 Å and *c* = 174.2 Å, α = β = γ = 90°. The asymmetric unit of the crystal contained one AlaA dimer with a solvent content of 43%.

**Table 2:** Crystallographic data processing and refinement statistics of AlaA.

	AlaA
**Data processing**	
Beamline	ID29 (ESRF)
Wavelength (Å)	0.97618
Resolution (Å)	46.88-2.11 (2.22-2.11)^a^
Space group	*C*222_1_
Unit cell dimensions	
*a*, *b*, *c* (Å)	59.78, 152.13, 174.24
α = β = γ (°)	90
R*merge*	0.081 (0.674)
R*meas* b	0.087
R*pim* b	0.030 (0.269)
CC_1/2_ c	0.999 (0.671)
CC*^c^	1.000 (0.896)
Total number of observations	367,856 (47,870)
Total number of unique observations	46,169 (6,678)
Mean((*I*)/σ(*I*))	14.1 (3.0)
Completeness	99.9 (100.0)
Multiplicity	8.0 (7.2)
**Refinement**	
Resolution (Å)	46.16-2.11 (2.18-2.11)
Total number of reflections	46,114
Reflections in test set (%)	2,491 (5.4%)
R*work*/R*free*	0.2036/0.2466
*B*-factor (Å^2^)^d^	
Protein	56.69/47.72
LLP	43.70/36.50
Acetate	50.00/60.90
Solvent	46.15
R.m.s. deviations	
Bond length (Å)	0.005
Bond angle (°)	0.830
R.m.s.d. Δ*B* (Å^2^)^e^	3.886/4.131/3.735/3.341/7.967
Ramachandran plot	
Favored	96.00% (776 residues)
Allowed	4.00% (32 residues)
Outliers	0.00% (0 residues)
All-atom clashscore	5.80
**Structure**	
Monomers/ASU	2
Protein residues	1-404 (chain A), 2-405 (chain B)
Cofactor/Ligand	2 LLP (Lys-PLP)/2 Acetate
Water/Glycerol	148/1

a Highest resolution shell is shown in parentheses.

b R*meas* = Σ*_ηκλ_* (n/n−1)^1/2^ Σ*_i_* |*I_i_*(*hkl*)−<*I*(*hkl*)>|/ΣΣ*_ι_ I_i_*(*hkl*); R*pim* = Σ*_ηκλ_* (1/n−1)^1/2^ Σ*_i_* |*I_i_*(*hkl*)−<*I*(*hkl*)>|/ΣΣ*_ι_ I_i_*(*hkl*); where *i* is the *i*th measurement of reflection (*hkl*) and <*I*(*hkl*)> is the average over symmetry related observations of a unique reflection (*hkl*).

c CC_1/2_ is the Pearson correlation coefficient calculated between two random half data sets; CC* is the CC of the full data set against the true intensities, estimated from CC* = [2 CC_1/2_/(1+CC_1/2_)]^1/2^.

d *B*-factors shown for chains A and B (except water molecules).

e R.m.s. deviations on *B*-factors for bonded atoms (all protein atoms/main chain-main chain atoms/side chain-side chain atoms/main chain-side chain atoms) and for non-bonded atoms; calculated with MOLEMAN2 [59].

### Structure determination and refinement

The X-ray data were phased by molecular replacement with the CCP4 [28] program *AMoRe* [29] using a search model based on an aminotransferase from *Pyrococcus furiosus* (PDB 1xi9) [2] with 40% sequence identity with AlaA. The search model was modified such that side chains of conserved residues were kept, those of non-conserved amino-acid residues were trimmed to the Cγ atom [30] and the *B*-factor for the entire structure was set to the average Wilson *B*-factor of the data (41.3 Å^2^). The structure was iteratively built using *Coot* [31] and refined with REFMAC5 [32] and phenix.refine [33] to convergence with a final Rwork/Rfree of 0.2036/0.2466 and satisfactory geometry. The structure was validated with MolProbity [34] and deposited in the PDB with code 4cvq. Refinement and validation statistics are summarized in **Table 2**. All figures of protein structures were prepared with PyMOL (http://pymol.sourceforge.net).

### Homology modeling of AlaC and AvtA

Since the crystal structures of *E. coli* AlaC and AvtA have not yet been determined, we attempted to produce high-quality atomic models using established homology modeling methods starting from known crystal structures of closely homologous proteins. AlaC was modeled from a probable aminotransferase from *P. aeruginosa* (PDB 2×5d) with 92% sequence identity [35] and AvtA from a valine-pyruvate aminotransferase from *S. typhimurium* LT2 (PDB 3g7q) sharing 93% sequence identity (unpublished). Both structures lack the N-terminal 15–20 amino acid residues, hence modeling of this segment was not attempted. More importantly, the cofactor status of those crystal structures was either mixed (unlinked PLP or PMP) for *Pa*AlaC (**Figure S3** in **File S1**) or empty for *St*AvtA, thus restricting the usefulness of these structures in understanding their reaction mechanism. To overcome this limitation, we modeled the active sites in the same competent configuration seen in the AlaA crystal structure, characterized by the covalent attachment of the catalytic lysine to the PLP cofactor. Given the high degree of sequence identity between the available structural templates and the target sequences, we threaded the correct *E. coli* sequences onto the templates using Modeller 9.10 [36] and then selected the best model out of 500 independent models on the basis of the Z-DOPE normalized scores, which were −1.764 for AlaC and −1.978 for AvtA (a Z-DOPE score of less than −1 indicates a reliable model whereby 80% of the Cα atoms are within 3.5 Å of their correct positions). These intermediate models were then refined and minimized using the force field and Monte Carlo sampling methods implemented in Rosetta [37], [38] and the improved models were sorted and clustered to obtain a final model. The definitive AlaC and AvtA models showed correct stereochemistry as assessed by MolProbity [34].

### Measurement of generation times

Doubling times were estimated in each of the four designated media using static or aerobic cultivation (File S1). Doubling times (*g*, expressed in h) were calculated by monitoring OD_600_ of subcultures inoculated at an initial cell density of 0.05 over 24 h. OD_600_ values from 3–6 independent experiments were fitted to equation 1 and used to derive the associated standard error of the mean (SEM).
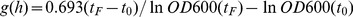
(1)


### Fitness experiments

The relative fitness advantage conferred by *alaA*, *alaC* and *avtA* gene products was assessed by running growth competition experiments between the wild-type strain (WT) and each of the corresponding single-gene deletion mutant strains. 2-mL co-cultures of WT and mutant strains were seeded equally using initial 10^−4^ dilutions from the two single strain overnight cultures in LB medium. The co-cultures were grown for ten days at 37°C with vigorous shaking and with daily back-dilutions of either 1∶10,000 (13.29 generations/day) or 1∶100,000 (16.61 generations/day). Cell concentrations were determined by counting colony-forming units (CFUs) on nonselective (LB) medium and selective (LB supplemented with 50 µg/mL kanamycin) medium. All data points were used to best fit exponential lines to determine slopes, which were divided by the number of generations to calculate the average growth rate differences of the mutants strains (File S1).

## Results

### Overall architecture

**Figure 2:**
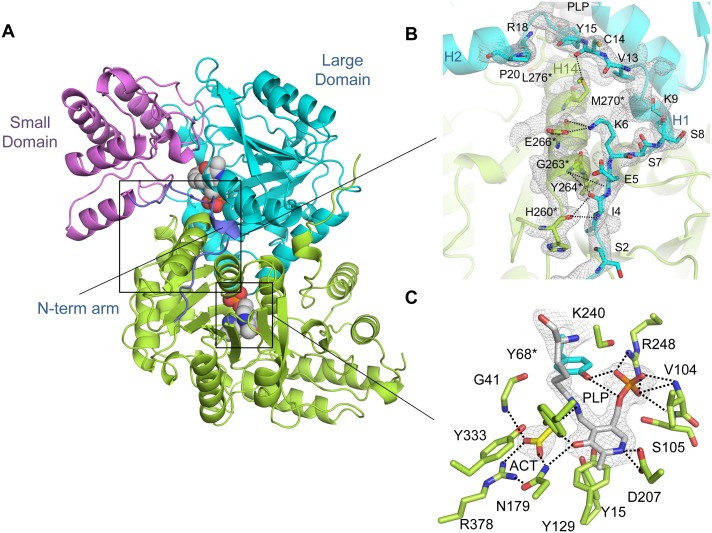
Overall and active site structure of *E. coli* AlaA in complex with acetate. (**A**) Ribbon representation of the overall structure of AlaA. One of the chains is shown in green while the other is shown in domain colors: the central, large domain in cyan, the small domain in violet, and the N-terminal arm of both chains is shown in deep blue. The PLP cofactor is shown in spheres and CPK colors. (**B**) Detail of the interaction between the N-terminal arm (H1-plug-H2) motif of one AlaA subunit (green) with helix H4 from the other subunit (cyan), shown with the experimental σ_A_-weighted electron density map (2*m*Fo–DFc) contoured at 1.0 σ level. Relevant helices and residues that participate in the interaction are labeled. (**C**) Inset of the active site. Active site residues are shown in sticks and color-coded as in (B); the carbon atoms of Lys240-PLP are in grey and in yellow in the acetate anion. Polar interactions within 3.5 Å of the PLP cofactor and acetate are represented by dotted lines. The experimental σ_A_-weighted electron density map (2*m*Fo–DFc) contoured at 1.0 σ.

The crystal structure of full-length AlaA from *E. coli* was determined by molecular replacement to 2.11 Å resolution and showed an overall structure that shares the distinctive features of fold type I aminotransferases [9], [39], characterized by a symmetric α_2_ homodimer with two identical composite active sites (**Figure 2A**). The 405-amino-acid residue AlaA monomer consists of a three-layered (α/β/α) large domain (residues 40–346) wedged between an N-terminal arm (amino acids 1–39) and a (α/β) C-terminal lobe (residues 303–405), whose structural integrity is maintained by a zipping interaction between two adjacent β-α motifs. The active site sits over the C-terminal face of the central parallel β-sheet of the large domain at the intersection between the large domain, the small domain and the N-terminal extension (**Figure 2B, C**). The dimerization interface is constructed from the first ten residues of the N-terminal arm and several α-helices from the flank and the base of the large domain, and is crucial for the organization of a functional active site since a catalytic tyrosine residue is donated from the opposite subunit (Tyr68*, where the asterisk denotes the second subunit). The area of the dimerization interface is 2973 Å^2^ and includes 43 hydrogen bonds and 4 salt bridges. Analysis of the relative orientation of the large and small domains and the presence of interpretable electron density for the complete length of the N-terminal segment (including the first methionine residue) suggest that AlaA was crystallized in the closed form [15], [40], [41].

Besides its contribution to shaping the dimer interface, the N-terminal arm of AlaA folded back onto the large domain and penetrated the active site cavity as an inwardly pointing U-shaped loop (or plug) (**Figure 2B**). In this conformation of the N-terminal arm, the side chains of Tyr15, Ile17 and Arg18 fall within hydrogen bonding or van der Waals distance of catalytic residues and, presumably, substrates and reaction intermediates. This four-residue stretch is bracketed between two helices, H1 and H2, which are mutually perpendicular and rest upon H14 from the large domain. A Gly19-Pro20 motif marks the end of the flexible helix H1-plug motif and the beginning of helix H2, which flanks the central (α/β/α) domain and is well ordered. Consequently, Pro20 acts as an anchor for the flexible N-terminal arm (**Figure 2B**).

### The active site of AlaA is poised for transamination

An intact Schiff base linkage between Lys240 and PLP and the presence of a substrate-mimicking acetate ion deeply buried in the active site indicated that the crystal structure of AlaA mimics the structure of the Michaelis complex with alanine (**Figure 2C**).

In addition to the constellation of electrostatic interactions that secures PLP binding through its phosphate moiety (including the side chains of Ser105, Ser239, Arg248 and Tyr68*, and the backbone amides of Val104 and Ser105), the covalent bond with Lys240 induces a strained conformation in PLP that enhances the reactivity of the cofactor and is further sustained by an extended network of polar and apolar interactions [42]. The degree of torsional strain of the internal aldimine in AlaA is considerable since the diagnostic C3-C4-C4’-Nζ dihedral angle is 77.7°, suggesting that the acidity of the Nζ-C4’ imine bond may have greatly increased (p*K*_a_ is probably 6.8–7.0) [42]. The precise orientation of the pyridinium ring is maintained by aromatic stacking with the side chain of Tyr129 together with hydrogen bonding of the O3’ atom with Tyr210 and Asn179 side chains. In particular, the conserved hydrogen bonding interactions between Tyr210 and the cofactor’s O3’ and Nζ atoms serve to modulate the Schiff base reactivity by further decreasing the p*K*_a_ of the imine and influencing the electron repartition during catalysis [42], [43]. In accord with the requirements for transaminase activity and control of reaction specificity [11], the acidic carboxylate group of Asp207 keeps the N1 atom of the pyridinium ring in a protonated state through a salt bridge interaction.

Binding of acetate (**Figure 2C**, ACT) is achieved by the concerted interaction of three highly conserved residues that are known to be essential for catalysis [44]. Acetate is mostly stabilized by a bidentate salt bridge interaction with the guanidinium group of Arg378 which mimics the binding mode and orientation of α-carboxylic substrates [39] (**Figure 2C**). In addition, the side chains of Asn179 and the main-chain amide nitrogen of Gly41 become engaged in the hydrogen-bonding network centered on the bound acetate in a manner analogous to a Michaelis complex. In particular, Asn179 and Arg378 participate in establishing the so-called PLP-N-R hydrogen bond linkage system between cofactor and substrate that is a hallmark of productive enzyme binding and poises the substrate for transamination [43]. Although acetate cannot react and therefore acts as a competitive inhibitor of alanine, its presence in the active site is associated with structural features of the closed conformation of aminotransferases capturing substrates or external aldimine complexes, such as a fully structured N-terminal arm and a sterically restricted active site cavity [15], [40], [41]. Hence, the carboxylate moiety of acetate may represent the minimal group capable of inducing (or selecting) a catalytically poised conformation. In fact, several residues coming from the plug motif (Tyr15 and Ile17) and branched aliphatic residues from both subunits (Ile40 and Leu276*) further constrict the active site pocket and reduce the accessible surface area to a mere 56 Å^2^ (**Figure S2** in **File S1**).

### Fully conserved active site among bacterial, archeal, plant and human alanine aminotransferases

**Figure 3:**
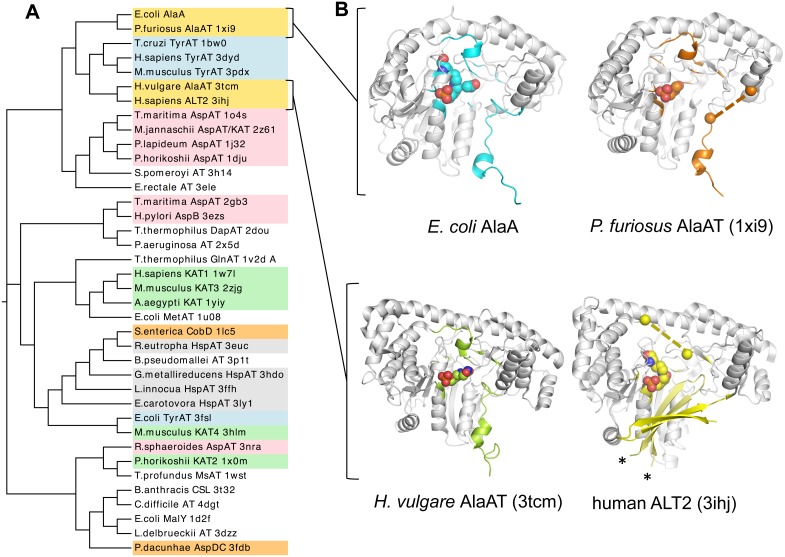
Comparison of AlaA with structurally homologous enzymes. (**A**) Phylogenetic tree based on structure-based multiple sequence alignments of AlaA obtained from PDBeFold [58]. Functionally related enzymes are shaded in like colors; alanine transaminases in gold, tyrosine aminotransferases (TyrAT) in cyan, aspartate aminotransferases (AspAT) in pink, kynurenine aminotransferases (KAT) in green, aspartate decarboxylases (CobD, AspDC, in orange), histidinol phosphate aminotransferases (HspAT) in grey; other transaminases of unknown function or with unique substrate preferences are not shaded. (**B**) Cartoon representation of alanine transaminases of known structure, highlighting the overall fold structure, catalytic residues, cofactor status and N-terminal motifs of AlaA (PLP, acetate), *Pf*AlaAT (PMP, PDB 1xi9), *Hv*AlaAT (DCS, PDB 3tcm) and human ALT2 (PLP, PDB 3ihj). In AlaA and *Hv*AlaAT the N-terminal H1-plug-H2 motifs are fully structured, whereas in *Pf*AlaAT and ALT2 different segments of the N-terminal arm are disordered. The most representative *Pf*AlaAT monomeric structure (present in three out of four copies in the crystal asymmetric unit) lacks interpretable electron density for the eight-residue segment (from Ala14 to Leu20, delimited by orange spheres) spanning the plug. In ALT2, the N-terminal 65-amino-acid residues fold into a long β-hairpin structure that swaps domain and extends toward the opposite subunit (the start and end of the swapped β-hairpin are marked with asterisks), partially covering the active site cavity and ending in a ten-residue unstructured segment (spanning Ile95 to Gln104 until the anchor Pro105 residue). This disordered region (delimited by yellow spheres) is also located over the substrate-binding pocket and therefore may have functional and structural roles akin to those of the plug motif described in AlaA and *Pf*AlaAT.

We queried the PDB for structural homologs of AlaA and retrieved about 40 crystal structures from widely different phylogenetic origin spanning bacteria, archea, protists, plants and mammalians (**Figure 3A**) with root-mean-square deviations (RMSD) ranging between 1.6–3.7 Å over 315–395 superposed Cα atoms. The closest structural homologs included the three *bona fide* alanine aminotransferases whose crystal structures have been determined to date (in gold, **Figure 3A**), several eukaryotic tyrosine aminotransferases and archeal and eubacterial aspartate aminotransferases. Among the former, structures of biochemically validated alanine aminotransferases from the archeon *P. furiosus* (*Pf*AlaAT, PDB 1xi9) and human ALT2 (PDB 3ihj) were solved by X-ray crystallography by various structural genomics consortia (unpublished), and the crystal structure from barley (*Hordeum vulgare*) alanine aminotransferase at 2.71-Å resolution was recently published as a cycloserine suicide complex (*Hv*AlaAT, PDB 3tcm) [45].

The global monomeric structures of all four alanine aminotransferases are similar (**Figure 3B**) despite their phylogenetic spread and the limited sequence similarity between them (26–44%). The AlaA structure can be superimposed with an RMSD of 1.60 Å (over 390 Cα atoms) with *Pf*Ala, 1.80 Å (over 395 Cα atoms) with *Hv*AlaAT, and 2.80 Å (over 366 Cα atoms) with hALT2. While hALT2 possesses a unique N-terminal segment that is folded as a β-hairpin and functions as a dimerization motif by associating with the opposite subunit (**Figure 3B**), the N terminus of AlaA, *Pf*AlaAT and *Hv*AlaAT comprises a flexible H1-loop motif (**Figures 2B** and **3B**). The anchor Gly-Pro dipeptide after the H1-loop described for AlaA is also found in *Pf*AlaAT and *Hv*AlaAT preceding helix H2, thereby indicating that the entire N terminus up to the anchor Gly-Pro sequence of alanine transaminases constitutes a conserved structural, functional and likely regulatory segment. A similar Pro anchor motif is also found in hALT2, where Pro105 serves to end a flexible region that extends from the β-hairpin dimerization segment into the active site. The disordered portion (from Ile95 to Gln104) is located over the substrate-binding pocket and therefore may have functional and structural roles akin to those of the plug motif described in AlaA and *Pf*AlaAT. In line with the conservation of the monomer structures, the dimerization interfaces of the four alanine aminotransferases span extensive interface areas (between 2281–3514 Å^2^) and include mostly polar interactions (31–44 hydrogen bonds and up to a maximum of 5 salt bridges).

**Figure 4:**
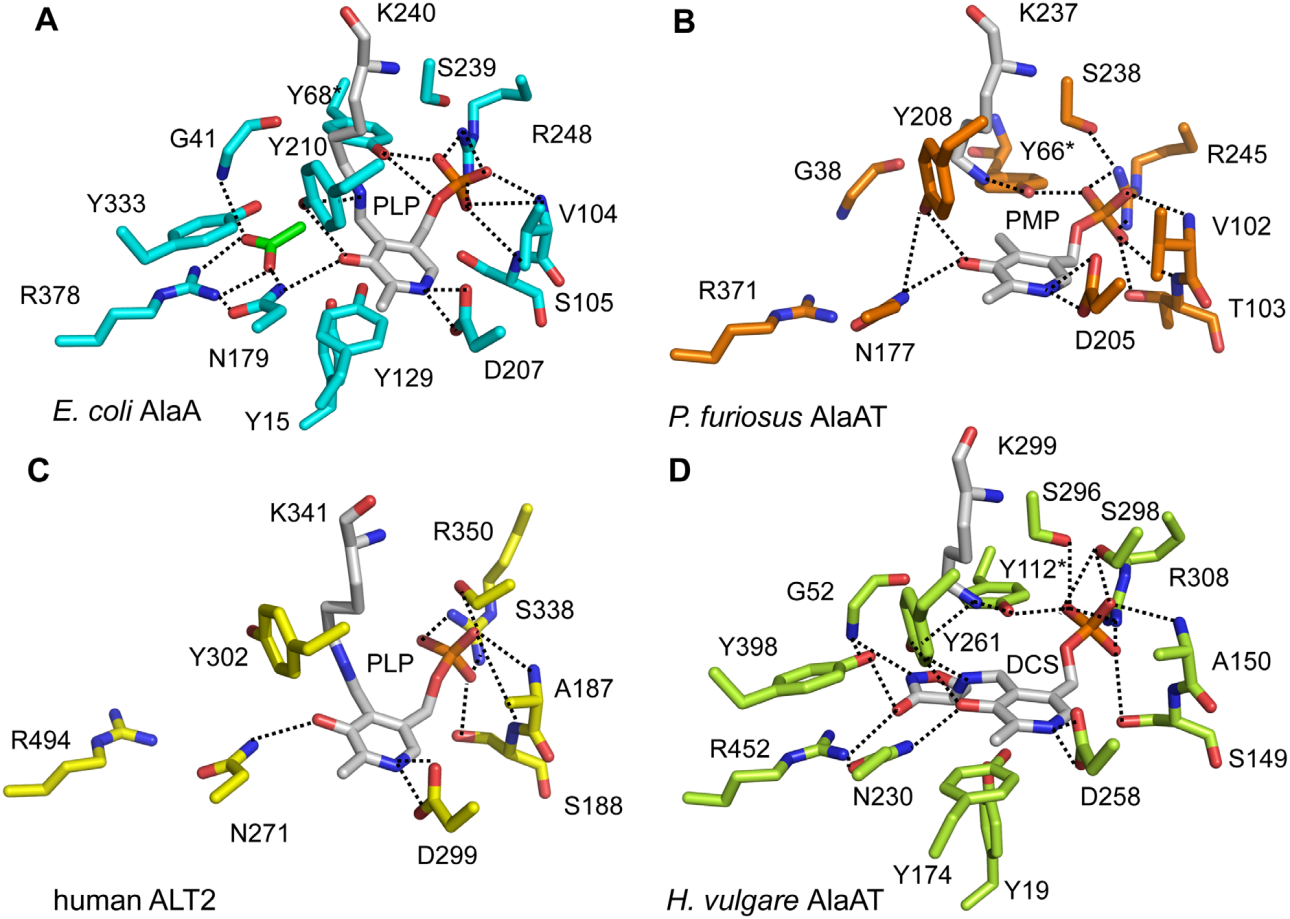
Active site architecture of alanine aminotransferases. Catalytically important amino acids and the cofactor of alanine transaminases: AlaA (**A**), *Pf*AlaAT (PDB 1xi9) (**B**), human ALT2 (PDB 3ihj) (**C**) and *Hv*AlaAT (PDB 3tcm) (**D**) are represented in sticks and color coded as in Figure 3. Dashed lines represent polar interactions. (A) Carbon atoms of the acetate anion are shown in green. (B) *Pf*AlaAT in the PMP form with a disordered N-terminal segment. The pyridoxamine ring of PMP is packed between Ile207 and Tyr127 (not shown for clarity). (C) In ALT2 all active-site residues pinpointed in AlaA (A) and *Pf*AlaAT (B) are structurally conserved, except for the residue equivalent to Gly41 (AlaA)/Gly38 (*Pf*AlaAT). Gly96 (ALT2), which falls in a disordered loop (Ile95 to Gln104), is a likely candidate to assume the substrate-binding role of Gly41 (AlaA) because of its proximity. (D) The suicide inhibitor complex of D-cycloserine (DCS) in the active site of *Hv*AlaAT assumes an identical configuration to that of AlaA, likewise accompanied by a fully ordered N-terminal motif that is closer in structure to the bacterial/archeal enzymes than to the human ALT2 despite the 44% sequence identity (by comparison, *Hv*AlaAT and AlaA are only 28% sequence identical).

In accord with the greater preservation of functional amino acid residues within homologous protein families, the active sites of the bacterial, plant and human enzymes share a conserved repertoire of catalytic residues that adopt equivalent positions and orientations, including the residues that in AlaA ligate the pyridoxal cofactor and the acetate (**Figure 2C** and **Table S1** in **File S1**). Indeed, very minimal discrepancies in primary sequence exist in the active site of the crystallized alanine aminotransferases and those few always involve conservative substitutions; e.g. Ser105 in AlaA is equivalent to Thr103 in *Pf*AlaAT (**Figure 4A, B**).

The available alanine aminotransferase structures captured different snapshots along the reaction coordinate; hence their comparison offers the possibility of evaluating changes in the active site during catalysis. While ALT2 was crystallized in the PLP form before substrate binding (**Figure 4C**), *Pf*AlaAT, with PMP in the active site (**Figure 4B**), represents the end state after the first half-reaction (**Figure 1**) after alanine conversion into pyruvate. In contrast, AlaA and *Hv*AlaAT structures are in complex with inhibitors (**Figure 4A, D**). We propose that the AlaA structure with an acetate anion bound in the active site corresponds to the Michaelis complex in that it mimics the main-chain atoms of alanine without engaging interactions of the substrate adduct not present in the substrate-bound enzyme (**Figure 4A**). Finally, the *Hv*AlaAT complex with D-cycloserine is analogous to the external aldimine (covalent substrate adduct) (**Figure 4D**) and has fully released the torsional strain present in the PLP form and the Michaelis complex of the enzyme. It is noteworthy that in AlaA and *Hv*AlaAT structures the complex N-terminal motifs are consistently structured over the active site and contribute interactions to the substrate mimic acetate and the DCS suicide adduct (**Figure 3B**), whereas in the *Pf*AlaAT and ALT2 structures the analogous segment is either disordered (**Figure 3B**) or, more rarely, partially structured (not shown).

### Alanine/2-oxoglutarate dual specificity switch is favored by a sterically restricted active site

Amino acid aminotransferases are promiscuous enzymes that transaminate a relatively wide range of amino and oxo acid substrates using 2-oxoglutarate/L-glutamate as the near universal second half-reaction substrate/product pair (**Figure 1**) [46]. Conserved substrate-binding residues across diverse amino acid aminotransferases bind the main-chain α-carboxylate common to all substrates. In contrast, the more diverse chemical nature of the side chain of the first and second half-reaction substrates often requires that the same binding pocket implement a dual specificity mechanism [47]–[50].

In AlaA, alanine specificity seems to be favored by sterically reducing the space available to accommodate the side chain of the substrate (**Figure S2** in **File S1**), such that binding of side chains bulkier than that of alanine could be either energetically expensive or yield a catalytically unproductive complex. This steric selectivity filter could severely restrict productive binding of branched aliphatic and aromatic amino acids such as phosphoserine and histidinol phosphate [48], [49]. Accordingly, *Pf*AlaAT has been shown to be poorly reactive toward these substrates [2].

**Figure 5:**
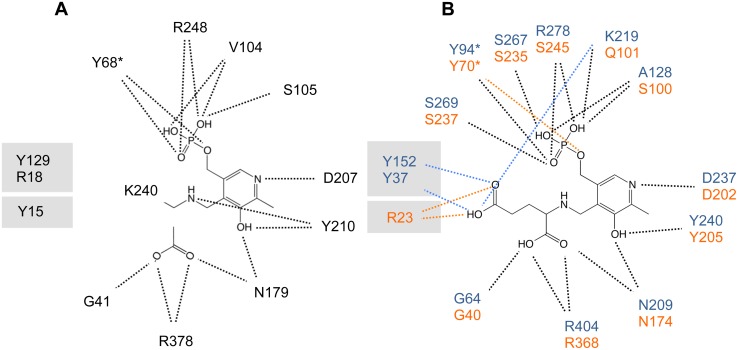
Dual substrate recognition in alanine aminotransferases: Catalytic pocket for dicarboxylic acid substrates. Schematic representation of the active site of AlaA in complex with acetate (**A**) and of *T. termophilus* α-aminoadipate transaminase LysN (PDB 2zyj) (**B**, orange) and *A. thaliana* LL-aminopimelate aminotransferase (PDB 3ei5) (**B**, blue) crystallized in complex with the glutamate external aldimine of PLP (PGU). AlaA residues Tyr15, Arg18 and Tyr129 (shadowed) are equivalent to residues known to stabilize the γ-carboxylate moiety of PGU, including Arg23 in α-aminoadipate transaminase and Tyr37 and Tyr152 in LL-aminopimelate aminotransferase.

A narrow active site, in turn, poses a steric challenge for the binding of the second half-reaction substrate, 2-oxoglutarate, whose entry would be seriously impeded by the confluence of multiple side chains in close proximity to the acetate anion in the AlaA crystal structure (**Figure 4A**). Steric clashes could occur with amino acids from the plug motif (Tyr15 and Ile17), from the same subunit (Ile40, Gly41 and Tyr129) and from the opposite subunit (Ile276*). Modest conformational rearrangements could however widen the active site cavity enough to accommodate the external aldimine of 2-oxoglutarate. The required structural changes could minimally entail an increased flexibility of the plug motif as that seen in the *Pf*AlaAT crystal structure, since a small displacement of Tyr15 and Ile17 side chains would create sufficient room to accommodate the side chain of 2-oxoglutarate. Additional electrostatic interactions with the Tyr15 hydroxyl group or the guanidinium side chain of Arg18 could further stabilize the γ-carboxylate of 2-oxoglutarate. Indeed, superposition of AlaA with α-aminoadipate transaminase LysN from *T. termophilus* (PDB 2zyj) [51] and with *A. thaliana* LL-diaminopimelate aminotransferase (PDB 3ei5) [52], which were crystallized with N-(5′-phosphopyridoxyl)-L-glutamate (the external aldimine of glutamate), shows that the side chain of Arg18 in AlaA is already very close to the expected location for the γ-carboxylate and could therefore stabilize its negative charge (**Figure 5**).

Taken together, the flexibility of the N-terminal plug motif of alanine aminotransferases and several conserved catalytic residues seem sufficient to implement a dual specificity mechanism capable of distinguishing between L-alanine/pyruvate and 2-oxoglutarate/L-glutamate. The dual specificity shift would only require subtle main chain and side chain rearrangements to elicit the switch of specificity.

### Alanine aminotransferase repertoire in *E. Coli*

**Figure 6:**
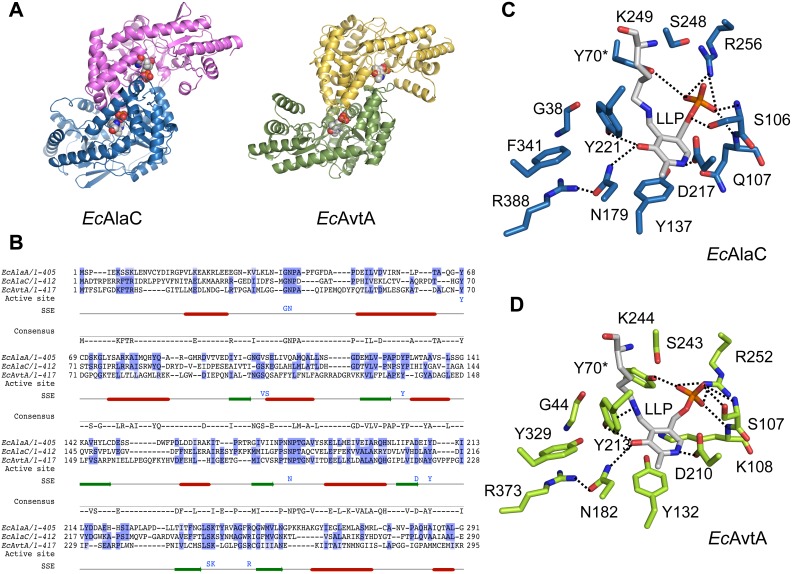
Homology models of the AlaC and AvtA alanine aminotransferases from *E. coli*. (**A**) Overall architecture of the homology models of *E. coli* AlaC (left) and AvtA (right) shown in ribbon representation and chain colors; PLP is shown in spheres and CPK colors. (**B**) Sequence alignment of AlaA, AlaC and AvtA. Conserved positions are shaded in blue; consensus active site residues are listed in blue underneath the alignment; secondary structural elements are shown (helices in red and strands in green). Despite the low sequence identity between AlaC and AvtA when aligned with AlaA (21% and 23%, respectively), the residues of the active site are strictly conserved with exception of Val104 and Ser105, which interact with the phosphate group mainly through backbone atoms. (**C–D**) Modeled active site configuration of AlaC (C) and AvtA (D). While the global fold was modeled on the basis of the closest bacterial homologous structures, the position and orientation of the PLP cofactor, which is crucial for catalysis, was modeled after the structure of the AlaA active site.

Alanine transamination in *E. coli* is catalyzed by at least eleven distinct transaminases, of which the three predominant enzymes are AlaA, AlaC and AvtA [17]. Their low sequence identity (below 25%) (**Figure 6B**) conceals a considerable degree of conservation within the active site. In agreement with the known relaxed substrate specificity of these enzymes, this conservation serves to generate the necessary biological redundancy that safeguards alanine and pyruvate homeostasis. While AlaA and AlaC are considered exclusively alanine aminotransferases, AvtA is a valine-pyruvate aminotransferase [17], [19]. Recently, crystal structures of AlaC and AvtA have been described for closely homologous enzymes (∼93% sequence identity) from *Pseudomonas aeruginosa Pa*AlaC (PDB 2×5d) [35] and *Salmonella typhimurium* LT2 *St*AvtA (PDB 3g7q) (unpublished). However, none of these homologous structures describe a catalytically competent active site since the cofactor is either missing, as in *St*AvtA, or in PMP or unlinked PLP forms, as in *Pa*AlaC. In the latter cofactor complexes, the orientation of the cofactor was reversed from that of a canonical PLP active site and with a solvent phosphate ion located in the canonical phosphate moiety of the cofactor-binding pocket. This altered active site configuration is likely to interfere with the establishment of a proper PLP-N-R hydrogen bond linkage system upon substrate binding, thereby impairing enzymatic activity (**Figure S3** in **File S1**). To overcome these shortcomings of the available homologous structures, we constructed homology models for the corresponding *E. coli* enzymes in the PLP form and used them for comparison with the AlaA structure (**Figures 6A**).

The high sequence similarity between the model and template sequences led to global folds similar to those of the template structures (**Figure 6A**). Likewise the high degree of conservation in the active site residues of the model sequences and AlaA is responsible for an almost identical chemical landscape for the PLP binding residues. Based on the models, AlaA, AlaC and AvtA shared the same set of residues for binding the phosphate group and pyrimidine ring of the cofactor (Tyr129, Tyr210 and Arg378) (**Figure 6C, D**). However, major variability is observed in the residues that interact with the phosphate group through main-chain groups, in the substitution of Tyr333 by Phe in AvtA, and in the residues that participate in binding the second half reaction’s substrate, depending ultimately on the particular enzyme specificity (**Figure 6C, D**). In the case of AlaA and AlaC, Tyr15 and Arg18 stabilize the γ-carboxylate group of the incoming glutamate (**Figures 4A** and **6C**). In contrast, AvtA, which lacks tyrosine residues in the N terminus, could use the disordered Arg12 for this role (**Figure 6D**). These differences may make AvtA the least efficient of the three major alanine aminotransferases in *E. coli* [17] despite the high degree of sequence conservation in the active site.

### AlaA and AlaC play differential cellular roles in global GPT activity

To assess the relative importance of the three major bacterial alanine aminotransferases (AlaA, AlaC and AvtA) in the adaptation to conditions resembling the intestinal environment, we measured the doubling times using four different media under anaerobic conditions and conducted fitness or competition experiments between three single-gene KO strains and the isogenic WT strain BW25113. In the KO strains the deleted gene had been substituted by a selectable kanamycin-resistant phenotype that can be used to differentiate WT from KO strains. We hypothesized that a loss of fitness caused by the deletion of any of the alanine aminotransferase genes in the KO strains would translate into longer doubling times and a tendency to be outcompeted by the WT strain. Measurement of the doubling times on each tested media facilitated the interpretation of the competition experiments by providing a quantitative expectation for the null hypothesis since the total number of colonies should exclusively depend on the relative doubling times in the absence of interference or correlation effects. Fitness reduction was quantified by plating serial dilutions of the respective co-cultures, and results were expressed as percent KO colonies with respect to WT colonies.

**Figure 7:**
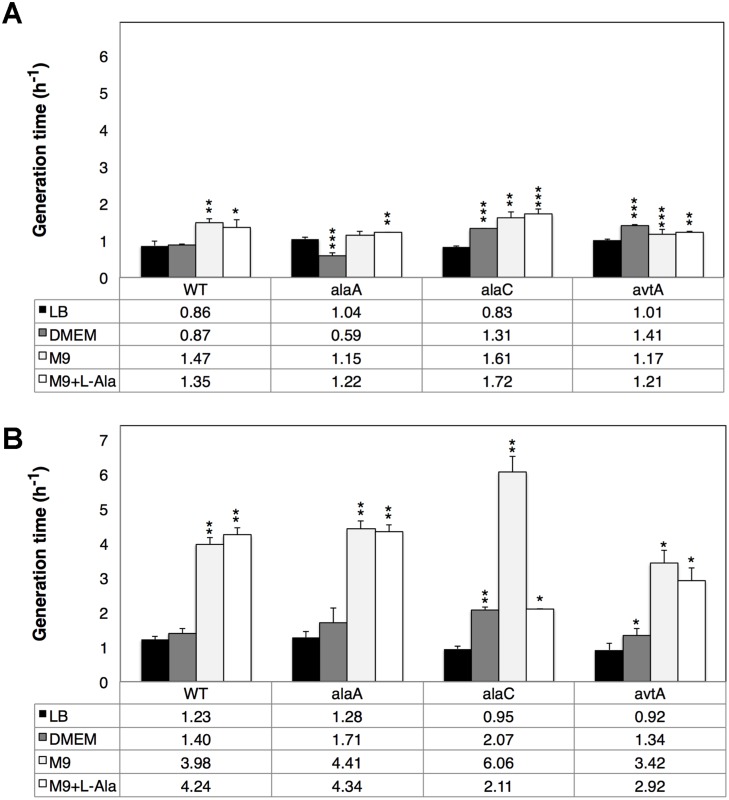
Effect of cultivation parameters on generation times of *E. coli* K-12 (wild-type), Δ*ala*A, Δ*ala*C and Δ*avt*A strains. Histogram summarizing the generation times of *E. coli* K-12 (wild-type strain, WT) and L-alanine aminotransferase single-gene knock-out (KO) strains (Δ*ala*A, Δ*ala*C and Δ*avt*A) in different media under aerobic conditions (with shaking) (**A**) or under oxygen-limiting conditions (static cultures) (**B**). Error bars are standard errors from the mean (SEM) derived from *N* independent assays (*N* = 3–6). Statistical significance of differences between the generation times of WT and KO strains (under the null hypothesis of no difference) is assessed using a one-tail t-Student test for paired data and is depicted atop the bars according to *P*-value (*P*>0.05, no label; *P*<0.05, *; *P*<0.01, **; *P*<0.001, ***).

Doubling times were measured both with shaking (**Figure 7A**) and in static cultures (**Figure 7B**). As previously reported [17], under aerobic conditions doubling times for the WT and the three KO strains did not differ significantly in M9 minimal medium alone or supplemented with 10 mM L-alanine nor did they differ in rich LB medium. However, when WT and KO strains were assessed in DMEM, significant changes were noticeable in mutant doubling times with respect to the WT (**Figure 7A**). Signs of slower growth were more pronounced in Δ*alaC* and Δ*avtA* KO strains, with doubling times nearly twice as long as the WT (*P*<0.001), whereas the Δ*alaA* KO strain accelerated its division rate by 30% (*P*<0.01). It was particularly telling that under aerobic conditions (with shaking) no strain exhibited better growth in L-alanine-supplemented media compared with the respective non-supplemented media, thereby indicating that aerobic metabolism can compensate for a limited supply of L-alanine precursors. Static growth conditions, which can arguably mimic the intestinal environment better than oxygen-saturated cultures, had more dramatic consequences for the generation time and relative behavior of WT and KO strains (**Figure 7B**). Across all strains analyzed, doubling times in M9 minimal medium were considerably longer (often more than twofold) than in LB or DMEM. This trend reached its maximum in the Δ*alaC* KO strain, which had a six-fold longer generation time in M9 than in LB. The Δ*alaC* deletion mutant also showed extreme behavior in M9 medium in that it was the only strain whose division rate returned to DMEM levels (only twice longer than in LB) when L-alanine was added, while the doubling times of the remaining strains were largely insensitive to the presence of absence of the L-alanine supplement (**Figure 7B**). In summary, assessment of doubling times of WT and single-gene KO strains showed that bacterial adaptation under the static growth conditions thought to prevail in the human intestine was more sensitive to differences in genome-encoded fitness characteristics and nutrient availability than in aerobic environments.

**Figure 8:**
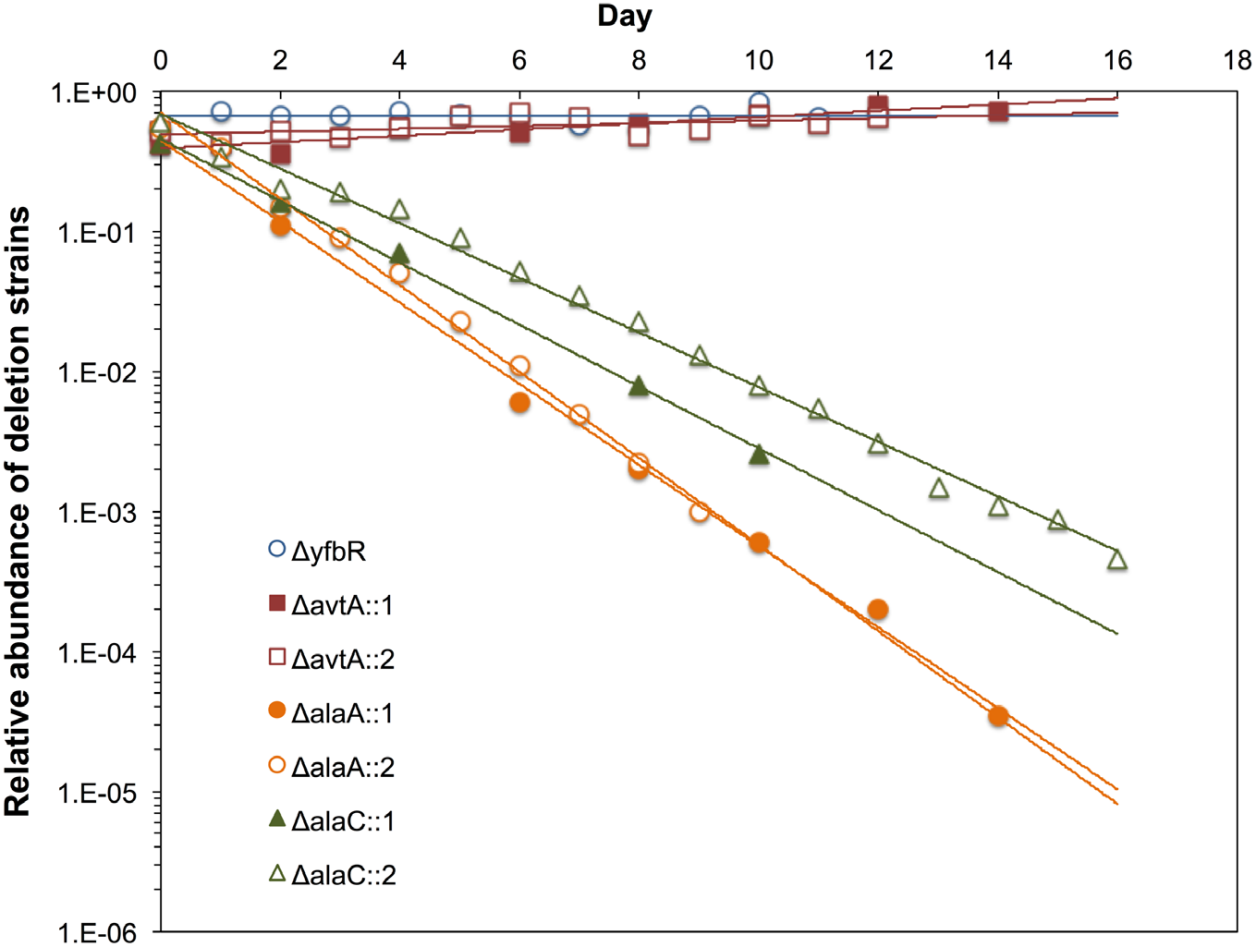
Growth competition between Δ*alaA*, Δ*alaC* and Δ*avtA* deletion mutants and the wild-type strain. The ratio of mutant to total (mutant plus WT) bacterial cells is plotted against time (in *d*) for two independent experiments. In experiment 1 (open symbols) the co-cultures were dialy back diluted to 1∶10,000 (13.29 generations/day) and experiment 2 (filled symbols) to 1∶100,000 (16.61 generations/day). Data points were used to best fit exponential lines to determine slopes, which were divided by the number of generations to calculate the average growth rate differences of the mutant strains. The starting ratios are indicated by the y-intercepts.

Growth competition experiments provide a more direct measure of relative fitness by co-cultivating the WT strain with each of the KO strains under defined conditions and counting the number of colonies per strain after a fixed number of generations (**Figure 8**). Both Δ*alaA* and Δ*alaC* mutations confer growth disadvantages when grown in rich media, as demonstrated by two separate competition growth rate experiments against the WT strain. In both experiments, Δ*avtA* deletion conferred no significant growth rate disadvantage indicating that under these experimental conditions the *avtA* gene is dispensable. Polar effects on downstream genes of the uncured kanamycin cassettes were assessed by inspection of the corresponding gene neighborhood maps [53]. While for the monocistronic Δ*avtA* and Δ*alaC* mutations polar effects were not possible, a potential polar effect was ruled out for the bicistronic Δ*alaA* mutation by performing a competition experiment using a mutation in the downstream *yfbR* gene (**Figure S4** in **File S1**).

Overall, these observations indicate that the relative fitness of the Δ*alaA*, Δ*alaC* and Δ*avtA* deletion strains when competing with the WT strain in rich medium with shaking was poorly predictable from their individual generation times, thereby indicating that a complex pattern of metabolic interactions might be at play. Importantly, measurement of generation times in static and aerated cultures and the results from the competition experiments show that even single-gene KO strains of the *alaA*, *alaC* or *avtA* genes, assayed under oxygen-saturated rich medium as well as under oxygen- or nutrient-limiting conditions, may have strong adverse effects on fitness and survival.

## Discussion

### A structural template for bacterial alanine aminotransferases

Since several different amino acid aminotransferases can catalyze the interconversion between alanine and pyruvate in bacteria from the microbiome, we set out to examine the shared structural and functional features that could explain their similarities and differences in the model organism *E. coli*. Although the structures of several such enzymes from other Gram-negative bacteria, like *Pa*AlaC and *St*AvtA, had already been determined, they were inappropriate for further analysis since the active site in these structures had either a mixed cofactor status, wrong orientation (*Pa*AlaC) (**Figure S3** in **File S1**) or was devoid of cofactor (*St*AvtA). In order to provide a sound basis for the structural and catalytic understanding of bacterial alanine transaminases, we determined the 2.11-Å-resolution crystal structure of *E. coli* AlaA in the PLP form in complex with the Michaelis complex analog acetate and constructed atomic-level homology models of *E. coli* AlaC and AvtA. These structural models and the available structures from archeal, plant and human enzymes provide insight into this large but poorly characterized class of enzymes.

The crystal structure of AlaA reveals traits that are presumably shared by the major bacterial alanine aminotransferases, AlaC and AvtA, and presumably by alanine aminotransferases of all life forms. Among these traits the following stand out prominently. First, a narrow, sterically restricted active site cavity that serves as the basis for alanine specificity during the first half-reaction and generates the need for a dual recognition mechanism in order to complete the catalytic cycle. Secondly, the crystal structure of AlaA and the other alanine aminotransferases possess an N-terminal segment with two distinct portions, a proximal, generally folded structure that participates in the dimerization interface and stretches over the top surface of the active site; and a distal U-shaped loop that we have termed the plug, 7–10 residues in length, including Tyr15, Ile17 and Arg18, which ends in a rigidly fixed Pro anchor residue followed by a helix. Whereas in archea and plants the plug motif is largely conserved in sequence and structure, the architecture of the active site of human ALT2 is different in that the stabilizing role exerted by Tyr15 on the glutamate adduct could potentially be taken over by the main-chain carbonyl groups of residues from the long and rigid N-terminal β-hairpin (Leu50* and Glu51*). Therefore, in human ALT2 the plug is located in a different side of the active center lying over Ile95 and Gly96, which are equivalent upon superposition to Ile40 and Gly41 in AlaA. In agreement with the essential role of the N-terminal sequences preceding the plug motif, a recombinant ALT2-2 isoform that lacks the first 100 residues (encoded by isoform 2 of *ALT2*) has been recently shown to be inactive [54]. The plug motif appears to become fully folded and/or completely stabilized only when a substrate or substrate complex analog is buried into the active site, since it has been observed in AlaA structure in complex with alanine-mimicking acetate, in a suicide inhibitor complex with *Hv*AlaAT, and in only one out of four asymmetric unit copies of crystalline *Pf*AlaAT in the PMP form. The combination of these two features, the flexible N-terminal arm and plug motifs and a restricted active site, must ensure proper substrate recognition and reaction selectivity since the variety of N-terminal motifs found so far in the structures of alanine aminotransferases are either very similar to that of AlaA or clearly functionally equivalent (ALT2).

### Proposed catalytic mechanism

The serendipitous finding of an acetate ion from the crystallization solution bound in the active site of AlaA permitted the precise mapping of the location and orientation of the alanine substrate and the interacting catalytic residues (**Figure 2C** and **4A**), and indicated the conformational changes that would be required for the productive binding of 2-oxoglutarate or L-glutamate. The strong salt bridge interaction between Arg378 and the acetate ion would secure alanine binding and properly position its α-amino group to become hydrogen bonded with PLP O3’ atom, therefore poised for the transaldimination reaction. At this stage, the sterically restricted active site of AlaA, delimited by the folded distal U-shaped loop from the N-terminal arm, Tyr210 and several aliphatic residues (e.g., Ile213, Leu332 and Leu382) can effectively discriminate between L-alanine and bulkier substrates. The strain inherent in the Schiff base linkage between Lys240 and PLP and the PLP-N-R hydrogen bond network [55] facilitate the formation of the PLP-alanine external aldimine and then helps orientate its Cα for proton abstraction by the side chain amine of Lys-240. The neighboring side chains of Tyr210 and Tyr68* (which are conserved in AlaC and AvtA) and, to a less extent, Tyr333 (which is conserved only AlaC, being a Phe in AvtA) may have catalytic roles in enhancing the reactivity of the Lys240 amine by increasing its acidity as well as in hydrogen bonding the imine of the external aldimine. The protonated side chain ammonium of Lys240 could then reprotonate the quinonoid PLP-alanine adduct at the C4’ position to generate a covalent adduct intermediate form (the ketimine form) (**Figure S1** in **File S1**), which is readily hydrolyzed by solvent water molecules. The high density of tyrosine residues in the immediate vicinity to the substrate pocket suggests that Tyr210 and perhaps Tyr129 could have additional catalytic roles in potentiating the nucleophilic character of incoming water molecules to favor ketimine hydrolysis. We propose that changes in the active site following adduct hydrolysis, such as the rearrangement of the N terminus, which would be consistent with the greater flexibility of the plug as seen in the *Pf*AlaAT structure with PMP, would facilitate the transit of incoming substrates between the two half reaction in the catalytic mechanism.

Comparison of the binding mode of acetate to AlaA with the closed structure of *E. coli* AAT [15] shows that AlaA has retained the essential residues Arg378 and Asn179 for binding of the common α-carboxylate function of the amino acid substrate while replacing the polar residues Ser296* and Arg292* from the opposite subunit of AAT, which stabilize the side-chain carboxylate of L-aspartate and 2-oxoglutarate, by Ile276* and Tyr15. In this manner, the dual specificity mechanism implemented in AlaA utilizes the branched aliphatic side chains of Ile17, Ile40 and Ile276* and the phenoxy side chain of Tyr15 to sterically sel­ect for L-alanine during the first half-reaction. Cofactor recycling requires the exit of pyruvate and subsequently the reaction of PMP with 2-oxoglutarate. Analysis of the AlaA crystal structure reveals that this shift in substrate specificity would be facilitated by the inherent flexibility of the N-terminal segment, which could easily accommodate the small side chain movements necessary to enlarge the active site cavity hence allowing access to 2-oxoglutarate. Stabilization of the dicarboxylic acid substrate could be accomplished by Arg18 from the U-shaped plug motif, which in the AlaA structure lies at an intermediate position from the two extreme Arg292* conformations in the the open and closed forms of AAT [15]. The side chain of Arg18 would require only minor conformational rearrangements of the sterically restricted active-site cavity to allow for the proper orientation and distance between the positively charged guanidinium group of Arg18 and the oppositely charged dicarboxylic acid. In addition, the phenoxy group of Tyr129 is suitably located in the vicinity of the substrate-binding pocket and could assist Tyr-15 in implementing the hydrogen-bonding network that achieves this charge shielding function. In fact, precedent has shown that fold type I aminotransferases in complex with a PLP-Glu covalent complex utilize either tyrosine or arginine residues (or a combination thereof) to stabilize the distal carboxylate moiety, often from the N-terminal arm and/or the PLP stacking tyrosine residue [41], [51], [52], [56], [57]. Tellingly, LysN, another aminotransferase of fold-type I, employs only Arg23 (PDB 2zyj) for this purpose [51] whereas LL-diaminopimelate aminotransferase (PDB 3ei5) exploits a tyrosine switch formed by Tyr37 and Tyr153 (equivalent to Tyr15 and Tyr129 in AlaA) [50].

There is a remarkable degree of structural and sequence conservation between AlaA and the homology models of AlaC and AvtA in spite of the low sequence identity between the three enzymes (below 25%). These conserved features could be better appreciated by correcting the cofactor status of the AlaC and AvtA homology models in analogy with the fully competent AlaA active site (**Figure 6C, D**). The tyrosine residues Tyr129 and Tyr210 occupy identical positions and have nearly identical chemical environments in the three L-alanine aminotransferases, thereby suggesting their involvement in common enzymatic tasks. Indeed, comparison of the active sites structures of AlaA, AlaC, AvtA, *Pf*AlaAT and ALT2 strongly suggest that these enzymes share a universal implementation of the transamination reaction mechanism.

In summary, the considerable degree of global and active site structural conservation across bacterial, archeal, plant and human alanine aminotransferases appears to be a necessary prerequisite for the maintenance of this central reaction in all life forms. Most divergence was found in the precise structural motifs employed to ensure efficient substrate binding and shield the reaction intermediates during catalysis. The few structures of distant AlaA homologs all have in common partially or completely disordered N-terminal segments that prevent detailed interpretations of the active site; in contrast, the equivalent motif in this new AlaA structure can be interpreted reliably in its entire length (including Met1). The structure of AlaA reported here in complex with a substrate mimic provides the first poised active site of an alanine aminotransferase (eukaryotic or prokaryotic), and provides evidence of the reorganization of a flexible segment between the catalytic residue Tyr15 and the conserved Pro20, which sets the limit of this mobile N-terminal region.

### Each alanine aminotransferase contributes differentially to overall fitness depending on environmental conditions

The reversible transamination of L-alanine to pyruvate is a central metabolic reaction whose relevance is stressed by the large number of enzymes capable of catalyzing this transformation. Single-gene deletion mutants of the genes that encode the major L-alanine aminotransferases in bacteria may not lead to a drastic reduction in fitness under aerobic conditions in a variety of experimental conditions [17]; however, these mild mutant phenotypes may not always hold for genetically heterogeneous bacterial populations growing aerobically on previously untested media or, more relevant to the physiologic situation, growing statically on the intestinal tract. While we have re-confirmed previous findings that doubling times of the Δ*alaA*, Δ*alaC* and Δ*avtA* single-gene deletion mutants in rich medium under oxygen saturation do not differ significantly from the WT, our experiments also reveal that other relevant but thus far untested conditions, e.g. DMEM medium, static growth, can render bacteria comparatively more sensitive to single-gene deletions affecting L-alanine transamination. In the latter regimes, strains lacking even a single gene can exhibit severe growth defects and an overall reduction of fitness, calling into question the assumption that the *alaA*, *alaC* and *avtA* genes have redundant functions.

A more remarkable observation is that competition experiments whereby the Δ*alaA*, Δ*alaC* and Δ*avtA* single-gene deletion mutants were challenged with the WT strain in rich medium and oxygen saturation already evinced drastic differences in fitness for the two major bacterial GPTs, AlaA and AlaC (in contrast, *avtA* is entirely dispensable without any adverse growth effect). This effect is comparatively more pronounced for the Δ*alaA* mutant than it is for the Δ*alaC* mutant, thereby suggesting that AlaA might have the greatest impact in bacterial cellular fitness among the pool of bacterial GPTs. Interestingly, the effects on fitness of the deletion mutations when compared to the WT strain cannot be simply accounted for by their respective doubling times, indicating that interference and competition processes are at play between the mutant and WT bacterial populations. Some of the observed effects may be qualitatively explained by assuming that *in vivo* the AlaA-catalyzed reaction is displaced toward pyruvate production from alanine whereas, conversely, the same reactions catalyzed by AlaC and AvtA favor alanine production from pyruvate.

These findings do not only highlight that caution should be taken when deriving conclusions about the relative survival of bacterial strains without their particular lifestyle (e.g., anaerobic *versus* aerobic environments) but also shed light on biological redundancy. In particular, these results illustrate that the network of compensatory effects brought about by the presence of several enzymes with partially overlapping functions may not be able to rescue some deletion phenotypes, therefore leaving adaptation and survival heavily dependent on environmental factors. This conclusion is in line with the systems biology view that biological redundancy does not provide backup functionality for the loss of enzyme activities, but rather increases the robustness of the metabolic network by maximizing net enzymatic activity over a spectrum of unpredictable and changing environmental conditions [21]. Hence, the manifold enzymes in bacteria that contribute to L-alanine transamination might have arisen during evolution from the pool of intrinsically promiscuous PLP enzymes in order to keep a steady-state level of L-alanine, pyruvate and related molecules in the face of changes in nutrient availability.

## Supporting Information

File S1 Combined Supporting Information File S1 contains Figures S1–S4 and Table S1. **Figure S1:** Detailed scheme of the reaction mechanism proposed for L-alanine transamination. **Figure S2:** Molecular surface representation of AlaA active site showing the highly sterically impeded binding pocket for substrate-mimic acetate ion. **Figure S3:** Active site configuration of the probable aminotransferase PA4715 from *Pseudomonas aeruginosa*) (PDB 2×5d) solved by the Scottish Structural Genomics consortium at 2.25-Å resolution. **Figure S4:** Gene neighborhood of *alaA*, *alaC* and *avtA* as depicted in EcoGene 3.0. **Table S1:** Active site interactions in crystallized L-alanine aminotransferases.
(PDF)

Data S1 Microbiology **r**aw data for Figures 7 and 8.
(XLSX)
