# Supplementary material for: Structural Analysis and Mutant Growth Properties Reveal Distinctive Enzymatic and Cellular Roles for the Three Major L-Alanine Transaminases of Escherichia coli
Source: PLoS One. 2014 Jul 11;9(7):e102139. doi: 10.1371/journal.pone.0102139 (PMC4094517; doi:10.1371/journal.pone.0102139)
Supplement: File S1 — Combined Supporting Information File S1 contains Figures S1–S4 and Table S1. Figure S1: Detailed scheme of the reaction mechanism proposed for L-alanine transamination. Figure S2: Molecular surface representation of AlaA active site showing the highly sterically impeded binding pocket for substrate-mimic acetate ion. Figure S3: Active site configuration of the probable aminotransferase PA4715 from Pseudomonas aeruginosa) (PDB 2×5d) solved by the Scottish Structural Genomics consortium at 2.25-Å resolution. Figure S4: Gene neighborhood of alaA, alaC and avtA as depicted in EcoGene 3.0. Table S1: Active site interactions in crystallized L-alanine aminotransferases. (PDF) [file pone.0102139.s001.pdf]

Supplementary Information

Structural analysis and mutant growth properties reveal distinctive enzymatic and cellular roles for the three major L-alanine transaminases of *Escherichia coli*

Esther Peña-Soler[1,2,#], Francisco J. Fernandez[1,#] , Miguel López-Estepa[1], Fernando Garces[4], Andrew J. Richardson[5], Juan F. Quintana[1], Kenneth E. Rudd[5], Miquel Coll[2,3] and M. Cristina Vega[1,*]

[1]Centro de Investigaciones Biológicas, Consejo Superior de Investigaciones Científicas (Spanish National Research Council, CSIC), Madrid, Spain.

[2]Institute for Research in Biomedicine (IRB Barcelona), Barcelona, Spain.

[3]Institut de Biologia Molecular de Barcelona (IBMB-CSIC), Barcelona, Spain.

[4]The Scripps Research Institute, La Jolla, California, USA.

[5]University of Miami Miller School of Medicine, Miami, Florida, USA

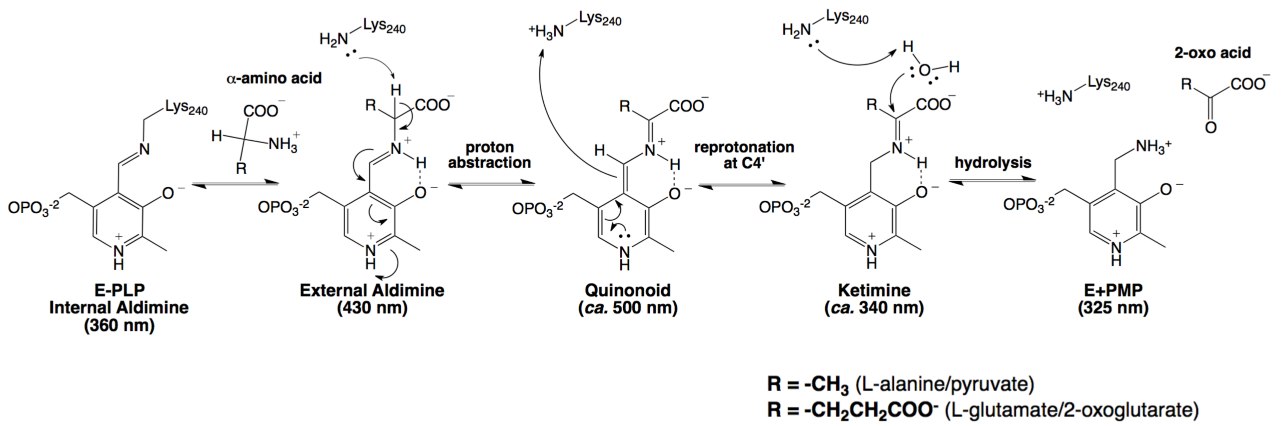

Supplementary Figure S1. Detailed scheme of the reaction mechanism proposed for L-alanine transamination. All L-alanine transaminases thus far characterized are pyridoxal 5'-phosphate (PLP, or vitamin B_6)-dependent enzymes of fold type I that catalyze the two sequential reactions that interconvert L-alanine and 2-oxoglutarate into pyruvate and L-glutamate, respectively (the amino acid / oxo acid substrate is shown with side chain **R**). In the case of valine-pyruvate transaminase (AvtA), the net reaction interconverts L-alanine and 3-methyl-2-oxobutanoate into pyruvate and L-valine. As such, both these enzymes are assumed to share a common enzymatic mechanism for L-alanine transamination that implies cofactor recycling from a covalently linked internal aldimine form of the cofactor (Lys-PLP) through a detached cofactor form as pyridoxamine 5'-phosphate (PMP). The first half reaction is initiated when the amino acid substrate reacts with Lys-PLP to yield a free lysine side chain and a covalent adduct between the PLP and the α-amino group of the incoming substrate, also called external aldimine. The substrate adduct is subsequently deprotonated at the Cα atom giving a resonance-stabilized quinonoid intermediate that is subsequently reprotonated at C4' yielding a ketimine, which is finally hydrolyzed to render as products the corresponding oxo acid (pyruvate from L-alanine and 2-oxoglutarate from L-glutamate; shown with side chain **R**) and PMP. This series of steps are universal for PLP-dependent aminotransferases. The regeneration of Lys-PLP from PMP takes place during the second half of the transamination reaction with glutamate as co-substrate and oxoglutarate as product. Factors such as the orientation of

the scissile bond and electron repartition in the resonance system of the covalent adduct are heavily influenced by the array of interactions established between the various cofactor forms and the active site residues, which ultimately determine the course of the reaction pathway toward transamination, decarboxylation, desulfination, elimination or aldol cleavage. The degree of versatility afforded by the chemistry of PLP-dependent enzymatic reactions has motivated the use as a scaffold for enzyme redesign and protein engineering studies of aspartate and aromatic amino acid transaminases.

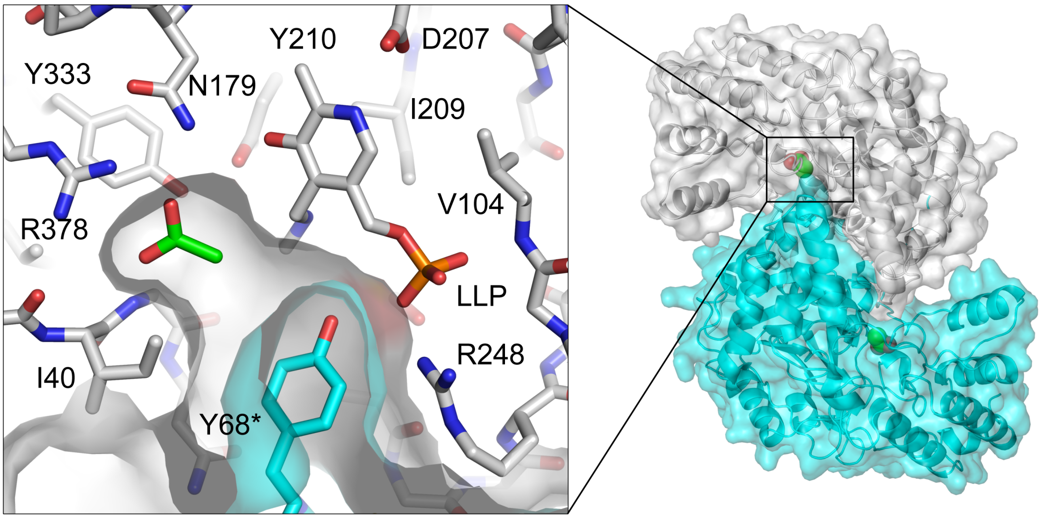

Supplementary Figure S2. The crystal structure of AlaA in complex with the substrate-mimic acetate reveals a highly sterically impeded complex indicative of a steric selectivity filter for amino acids with small side chains, since amino acid substrates with side chains slightly larger than L-alanine are likely to cause van de Waals clashes with active-site residues (e.g., Ile15, Ile40, and Ile276*). To illustrate this, we show a molecular surface representation of the AlaA dimer (where each chain is colored differently) and the acetate ion in space filling representation (in green). The inset on the left hand side shows a close-up on the acetate-binding pocket with the active-site residues delimiting the pocket walls and constricting the available exposed area.

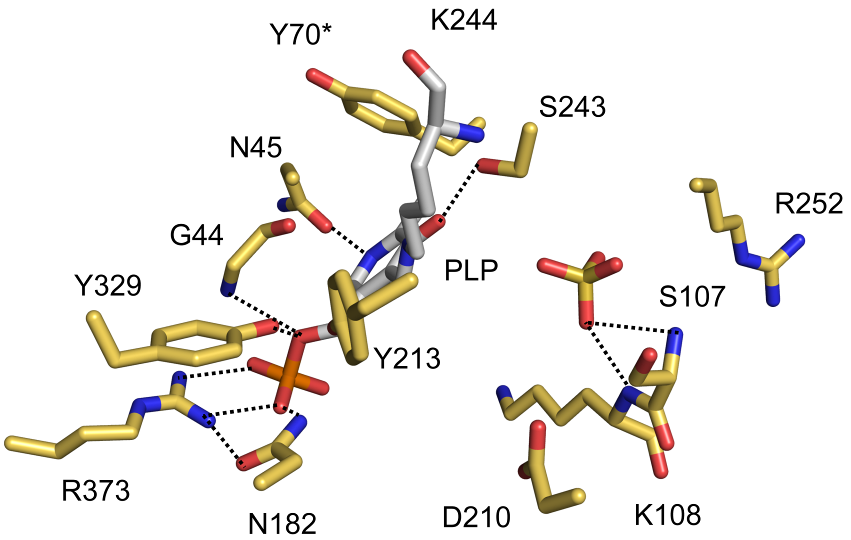

Supplementary Figure S3. Active site configuration of the probable aminotransferase PA4715 from *Pseudomonas aeruginosa*) (PDB 2x5d) solved by the Scottish Structural Genomics consortium at 2.25-Å resolution. The asymmetric unit contained four subunits arranged in two biological dimers (chains A–D and B–C). The authors stated that chains B and C lacked interpretable electron density for the cofactor and that sulfate ions were found in the active site instead. Chains A and D, however, contained interpretable density that was modeled as an unlinked PLP cofactor in chain D and as an internal aldimine of PLP with the catalytic lysine (Lys244) in chain A (shown). In both chains (A and D) the same orientation of either unlinked PLP or the internal aldimine is observed that is, however, different from the canonical orientation found in most PLP enzymes of fold type I and, in particular, different from the configuration that we had found in our crystal structure of AlaA (PDB 4cvq, this work). In most PLP enzymes, the pyridine ring of the cofactor has the *re* face toward the catalytic lysine (Lys244 in this case) and the phosphate moiety occupies a very conserved pocket involving highly conserved sequence motifs. However, the PLP cofactor in PDB 2x5d has flipped the orientation of its pyridine ring so that its *syn* face, rather than the *re* face, is now closest to the ε-amine group of Lys244. As a consequence of the rotation of the pyridine ring around the C1’-C4’ axis, the cofactor’s phosphate group is now located in a pocket across the active site and opposite to its canonical pocket. Instead, a sulfate ion has occupied this canonical location. Other

substrate-binding residues of the active site have also been altered by this unusual orientation of PLP, including, for example, Tyr329, Asn182 and Arg252. Tyr329 was expected to provide stacking interactions to hold the PLP in place, but in PDB 2x5d is freely standing while another tyrosine residue, Tyr132, partially compensates for its loss. The two residues Asn182 and Arg252 typically help organize the so-called PLP-N-R hydrogen bond network, which is instrumental for efficient catalysis; in PDB 2x5d, the bonding distances are perturbed and a working PLP-N-R is probably not formed.

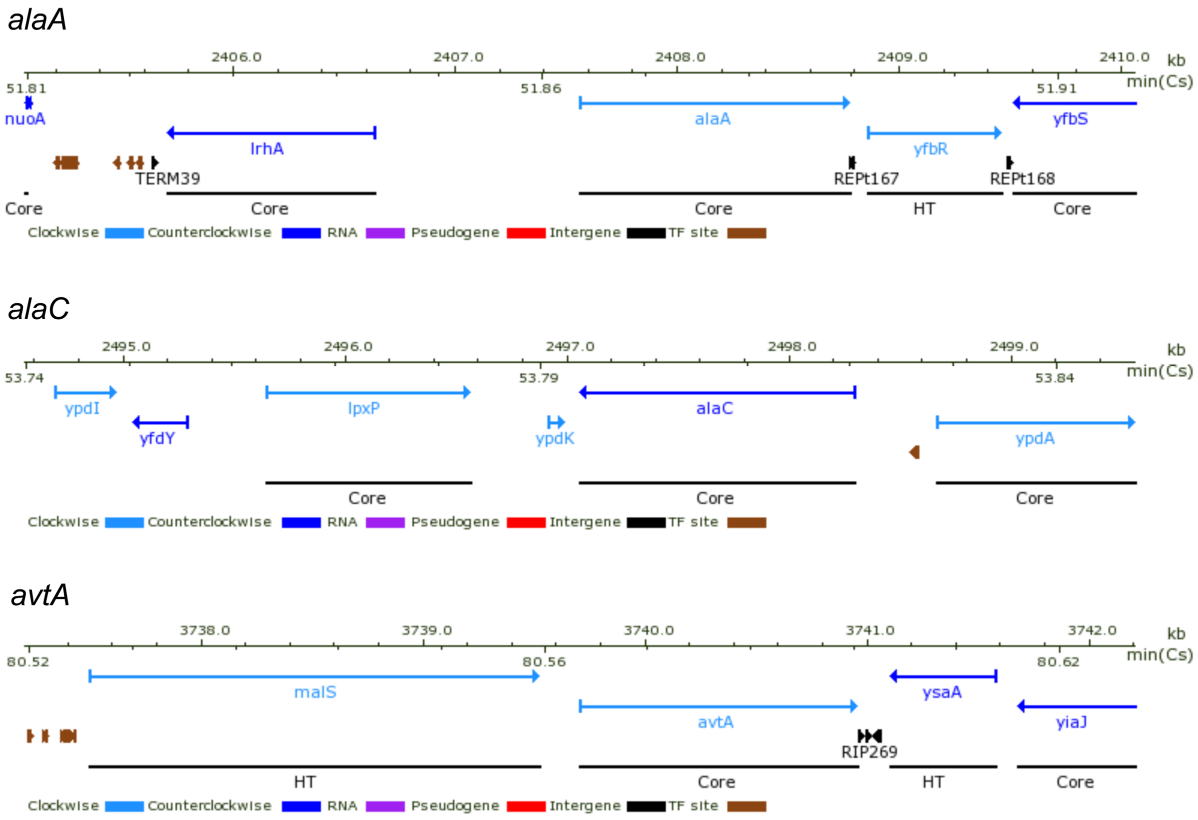

Supplementary Figure S4. Gene neighborhood of *alaA*, *alaC* and *avtA*. The genes and features around the three L-alanine transaminase genes as depicted in EcoGene 3.0 (Zhou J, Rudd KE (2013) EcoGene 3.0. Nucleic Acids Res 41: D613-624).

Supplementary Table S1. Active site interactions in crystallized L-alanine aminotransferases. An interaction is defined when the distance between a protein atom and a cofactor/substrate-mimic/inhibitor complex atom is < 3.5 Å. Polar and van der Waals interactions are both considered.

	**AlaA**	***Pf*AlaAT**<br>PDB 1xi9	***Hv*AlaAT**<br>PDB 3tcm	**ALT2**<br>PDB 1ihj
**Cofactor**[1]	Lys240	Lys237	Lys299	Lys341
	Tyr129	Tyr127	Tyr174	Tyr216
	Ile209	Ile207	Val260	Val301
	Tyr210	Tyr208	Tyr261	Tyr302
	Ser239	Ser238	Ser298	Ser338
	Arg248	Arg245	Arg308	Arg350
	Val104	Val102	Ala148	Ala187
	Ser105	Thr103	Ser149	Ser188
	Asp207	Asp205	Asp258	Asp299
	Asn179	Asn177	Asn230	Asn271
	Tyr68*	Tyr66*	Tyr112*	disordered
	n.eq.[3]	n.eq.[3]	Ser296	Ser338
**Alanine**[2]	Asn179	Asn177	Asn230	Asn271
	Arg378	Arg371	Arg452	Arg494
	Gly41	Gly38	Gly54	disordered
	Tyr333	Tyr328	Tyr398	Tyr440
	Tyr15	Tyr13	Tyr19	disordered

1 Cofactor refers to PLP (covalently linked to a catalytic Lys residue) and to PMP.
2 Substrate-binding amino acid residues. It also includes acetate-binding residues in AlaA and D-cycloserine-binding residues in *Pf*AlaAT.
3 n.eq. designates the absence of an equivalent residue. In AlaA, a water molecule from the solvent replaces the hydroxyl group of Ser296.
